# Remote assessment of ADHD in children and adolescents: recommendations from the European ADHD Guidelines Group following the clinical experience during the COVID-19 pandemic

**DOI:** 10.1007/s00787-023-02148-1

**Published:** 2023-02-11

**Authors:** P. Santosh, S. Cortese, C. Hollis, S. Bölte, D. Daley, D. Coghill, M. Holtmann, E. J. S. Sonuga-Barke, J. Buitelaar, T. Banaschewski, A. Stringaris, M. Döpfner, S. Van der Oord, S. Carucci, D. Brandeis, P. Nagy, M. Ferrin, D. Baeyens, B. J. van den Hoofdakker, D. Purper-Ouakil, A. Ramos-Quiroga, M. Romanos, C. A. Soutullo, A. Thapar, I. C. K. Wong, A. Zuddas, C. Galera, E. Simonoff

**Affiliations:** 1 Department of Child and Adolescent Psychiatry, Institute of Psychology, Psychiatry and Neuroscience, King’s College London, London, UK; 2 Centre for Interventional Paediatric Psychopharmacology and Rare Diseases, South London and Maudsley NHS Foundation Trust, London, UK; 3 HealthTracker Ltd, Gillingham, Kent UK; 4 Centre for Innovation in Mental Health, School of Psychology, Faculty of Environmental and Life Sciences, University of Southampton, Southampton, UK; 5 Clinical and Experimental Sciences (CNS and Psychiatry), Faculty of Medicine, University of Southampton, Southampton, UK; 6 Solent NHS Trust, Southampton, UK; 7 Hassenfeld Children’s Hospital at NYU Langone, New York University Child Study Center, New York City, NY USA; 8 Mental Health and Clinical Neurosciences, School of Medicine, University of Nottingham, Nottingham, UK; 9 School of Medicine, National Institute for Health Research (NIHR) MindTech Mental Health MedTech Cooperative, NIHR Nottingham Biomedical Research Centre, Institute of Mental Health, Mental Health and Clinical Neurosciences, University of Nottingham, Nottingham, UK; 10 Center of Neurodevelopmental Disorders (KIND), Centre for Psychiatry Research, Department of Women’s and Children’s Health, Karolinska Institute and Stockholm Health Care Services, Region Stockholm, Stockholm, Sweden; 11 Child and Adolescent Psychiatry, Stockholm Health Care Services, Region Stockholm, Stockholm, Sweden; 12 Curtin Autism Research Group, Curtin School of Allied Health, Curtin University, Perth, WA Australia; 13 NTU Psychology, School of Social Science, Nottingham Trent University, Nottingham, UK; 14 Departments of Paediatrics and Psychiatry, University of Melbourne, Melbourne, VIC Australia; 15 Murdoch Children’s Research Institute, Melbourne, VIC Australia; 16 Royal Children’s Hospital, Melbourne, Melbourne, VIC Australia; 17 LWL-University Hospital for Child and Adolescent Psychiatry, Ruhr University, Bochum, Germany; 18 Department of Child and Adolescent Psychiatry, Aarhus University, Aarhus, Denmark; 19 Department of Cognitive Neuroscience, Donders Institute for Brain, Cognition and BehaviourRadboudumc, Nijmegen, The Netherlands; 20 Karakter Child and Adolescent Psychiatry University Centre, Nijmegen, The Netherlands; 21 Department of Child and Adolescent Psychiatry and Psychotherapy, Medical Faculty, Central Institute of Mental Health, Mannheim/Heidelberg University, Mannheim, Germany; 22 Divisions of Psychiatry and Psychology and Language Sciences, University College London, London, UK; 23 First Department of Psychiatry, Aigineteion Hospital, National and Kapodistrian University of Athens, Athens, Greece; 24 Department of Child and Adolescent Psychiatry, Psychosomatics and Psychotherapy, Faculty of Medicine, University Hospital Cologne, University of Cologne, Cologne, Germany; 25 School of Child and Adolescent Cognitive Behavior Therapy (AKiP), Faculty of Medicine, University Hospital Cologne, University of Cologne, Cologne, Germany; 26 Clinical Psychology, KU Leuven, Louvain, Belgium; 27 Department of Biomedical Sciences, Sect. Neuroscience and Clinical Pharmacology, University of Cagliari, Cagliari, Italy; 28 Child and Adolescent Neuropsychiatry Unit, “A. Cao” Paediatric Hospital, ASL Cagliari, Cagliari, Italy; 29 Department of Child and Adolescent Psychiatry and Psychotherapy, University Hospital of Psychiatry, University of Zurich, Zurich, Switzerland; 30 Neuroscience Center Zurich, University of Zurich, Zurich, Switzerland; 31 ETH Zurich, Zurich, Switzerland; 32 Bethesda Children’s Hospital, Budapest, Hungary; 33 Barnet, Enfield and Haringey NHS Trust, ReCognition Health, London, UK; 34 Department of Child and Adolescent Psychiatry, University of Groningen, University Medical Center Groningen, Groningen, The Netherlands; 35 Department of Clinical Psychology and Experimental Psychopathology, University of Groningen, Groningen, The Netherlands; 36 Accare Child Study Center, Groningen, The Netherlands; 37 Unit of Child and Adolescent Psychiatry (MPEA1), CHU Montpellier-Saint Eloi Hospital, University of Montpellier, 80 Avenue Augustin Fliche, 34000 Montpellier, France; 38 Team Psychiatry, Development and Trajectories, INSERM CESP U 1018, University Paris Saclay, Gif-sur-Yvette, France; 39 Vall d’Hebron Research Institute (VHIR), Universitat Autònoma de Barcelona, Barcelona, Spain; 40 Department of Child and Adolescent, Center of Mental Health, University Hospital of Wurzburg, Wurzburg, Germany; 41 Louis A Faillace Department of Psychiatry and Behavioral Science, University of Texas (UT) Health, Houston, TX USA; 42 Division of Psychological Medicine and Clinical Neurosciences, MRC Centre for Neuropsychiatric Genetics and Genomics, Wolfson Centre for Young People’s Mental Health, Cardiff University School of Medicine, Wales, UK; 43 Department of Pharmacology and Pharmacy, The University of Hong Kong, Hong Kong, SAR China; 44 Research Department of Practice and Policy, UCL School of Pharmacy, London, UK; 45 University of Bordeaux, Bordeaux, France; 46 Bordeaux Population Health Centre, INSERM U1219, Bordeaux, France; 47 Child and Adolescent Psychiatry Department, Charles Perrens Hospital, Bordeaux, France

**Keywords:** ADHD, COVID-19, Remote assessment, European ADHD Guidelines Group (EAGG), Pandemic, Children, Adolescents

## Abstract

The COVID-19 pandemic led ADHD services to modify the clinical practice to reduce in-person contact as much as possible to minimise viral spread. This had far-reaching effects on day-to-day clinical practice as remote assessments were widely adopted. Despite the attenuation of the acute threat from COVID, many clinical services are retaining some remote practices. The lack of clear evidence-based guidance about the most appropriate way to conduct remote assessments meant that these changes were typically implemented in a localised, ad hoc*,* and un-coordinated way. Here, the European ADHD Guidelines Group (EAGG) discusses the strengths and weaknesses of remote assessment methods of children and adolescents with ADHD in a narrative review based on available data and expert opinions to highlight key recommendations for future studies and clinical practice. We conclude that going forward, despite remote working in clinical services functioning adequately during the pandemic, all required components of ADHD assessment should still be completed following national/international guidelines; however, the process may need adaptation. Social restrictions, including changes in education provision, can either mask or exacerbate features associated with ADHD and therefore assessment should carefully chart symptom profile and impairment prior to, as well as during an ongoing pandemic. While remote assessments are valuable in allowing clinical services to continue despite restrictions and may have benefits for routine care in the post-pandemic world, particular attention must be paid to those who may be at high risk but not be able to use/access remote technologies and prioritize these groups for conventional face-to-face assessments.

## Introduction

Attention-deficit/hyperactivity disorder (ADHD) is a common neurodevelopmental condition characterized by pervasive (present in more than one setting), impairing, and developmentally inappropriate symptoms of inattention and/or hyperactivity-impulsivity. ADHD typically begins during childhood and often persists into adulthood, although the manifestations may change, and the severity of hyperactivity can diminish with age. The prevalence of ADHD is estimated at 5–7% [43] in school-aged children and 2.5% in adulthood [58]. A wide range of other neurodevelopmental, psychiatric, and physical disorders often co-occur causing additional impairment [19]. Individuals with ADHD frequently experience difficulties with relationships, academic and vocational problems [1, 20]. Pharmacological treatments for ADHD can be highly effective in reducing core symptoms, at least in the short term. In addition, different behavioural or cognitive behavioural interventions tailored to the age of the person with ADHD are recommended by international clinical guidelines to address aspects beyond the core symptoms [12, 16]. Because ADHD is often a lifelong disorder with serious mental and physical impacts across the lifespan, and interventions can improve its course, early and accurate diagnosis is a public health priority [52, 60].

The COVID-19 pandemic led ADHD services to modify their practice and reduce in-person contact as much as possible. Clinicians had to rapidly adjust and adapt their usual practice to reduce the viral spread and protect the health of their child and adolescent ADHD patients, their families, other healthcare professionals and the broader community. This situation disrupted ongoing care for existing patients, and reduced access to services for new ones, during a period where lock-down restrictions may have exacerbated the difficulties experienced by people with ADHD [54, 57]. Furthermore, in many contexts and different countries, pre-existing delays in assessment and interventions in Child and Adolescent Mental Health Services (CAMHS) or Paediatrics were exacerbated by COVID-19 due to difficulties arising from increased demand and reduced availability of in-person assessments. To maintain services as best they could, clinical teams, where possible, turned to telemedicine/telehealth, with telephone and audio-visual appointments, to conduct assessments and consultations and used on-line tools for the assessment and monitoring of emotional, behavioural, and cognitive problems and functioning [11]. The urgent need to adopt remote approaches and to modify conventional assessments has had far-reaching effects on day-to-day clinical practice. However, there is a shortage of systematic evidence about which approaches are most feasible, reliable, accurate, and acceptable to patients and their families, and which are cost effective and time efficient. Guidance about the best way to work remotely was therefore lacking and changes were typically implemented in a localised ad hoc and un-coordinated way. In this article, the European ADHD Guidelines Group (EAGG) reflects on the practice of remote ADHD assessment during the COVID-19 pandemic and provides recommendations for best practice going forward. The EAGG was set up as a working group of the European Network for Hyperkinetic Disorders (EUNETHYDIS) in 2010 with the aim of providing clinical guidance to practitioners on the clinical management of ADHD based on a rigorous assessment of the evidence. The group currently includes 28 members (mainly child and adolescent psychiatrists or psychologists) with expertise in the pharmacological and/or non-pharmacological treatments for ADHD in children, adolescents, and adults. To date, the EAGG has published 13 papers in high profile journals. Additional information on the EAGG and its members is reported online at: https://eunethydis.eu/eunethydis-initiatives/european-adhd-guideline-group/.

We aim to highlight what we think are promising approaches as well as identify areas where remote assessments may currently be insufficient or unsatisfactory and where conventional approaches to assessment remain preferable. We also consider whether such approaches should continue to be used as part of routine care in a post-pandemic world. This is a rapidly developing area and so our goal is to present recommendations to assist remote ADHD assessment and research and provide examples of good practice and potential pitfalls.

## Impact of the pandemic on manifestations of ADHD and comorbid mental health problems

The impact of the COVID-19 pandemic on the mental health of children and adolescents, in general, appears to be multifaceted and substantial, with increases in anxiety and depression prevalence (rates of depression increased from pre-pandemic 2–6% to 12.9%) in a meta-analysis by Racine et al. [44], with higher rates in studies conducted later in the pandemic and in girls [44]. Individuals with ADHD are likely to be vulnerable to the confinements, changing rules and unpredictability caused by the COVID-19 pandemic [32]. The early observed increases in adolescent mental health symptoms during the COVID-19 pandemic do not, on average, appear to be sustained following the lifting of stay-at-home orders [45], though studies evaluating impacts on mental health across longer periods of time are needed [59]. Compared to the pre-pandemic period, children had less exercise, less outdoor time, and experienced less enjoyment in activities, while television, social media, gaming, sad/depressed mood, and loneliness were increased. Child stress about COVID-19 restrictions was associated with poorer functioning across most domains [53].

Systematic reviews [31, 39, 41] and longitudinal studies [29, 42], have estimated the impact of the pandemic on children with neurodevelopmental disorders. These groups are reported to have higher rates of mental health difficulties than those without pre-existing psychiatric disorders, due to COVID-19-related changes to daily life [41]. This finding is also supported by a survey of patients attending CAMHS at the South London and Maudsley NHS Trust, London, UK (Parlatini et al., personal communication). A longitudinal study, Co-SPACE, found a significant lock-down-related increase in symptoms of inattention and hyperactivity as well as behavioural problems in the general population in primary school-aged children and those with identified special educational needs or from low-income households, which were less likely to improve when schools opened [45, 56]. The study reported that in March 2021, as restrictions in the UK were eased, unlike the general child population, parents of children with special educational needs or neurodevelopmental disorders and those in low-income families continued to report high levels of symptoms [56].

Despite these observations, trajectories of ADHD, emotional, and behavioural symptoms during the periods of quarantine are less clear, with mixed and heterogeneous findings across studies and within study samples. For instance, a survey of parents of children and adolescents aged 3–20 years with ADHD early in the first lockdown in France showed an improvement in anxiety, less school-related problems and flexible schedules that adapted to the child together with improved self-esteem [6]. Another study of 238 adolescents aged 15–17 years (118 with ADHD), in which parents and adolescents provided ratings of mental health symptoms, found that early observed increases in adolescent mental health symptoms during the COVID-19 pandemic did not on average appear to be sustained following the removal of stay-at-home orders, but emotion dysregulation and ADHD increased the risk for sustained negative mental health functioning [7]. Another survey of 992 parents of children and adolescents aged 5–18 years with ADHD in Italy investigated the degree of severity of emotional/mood states and disrupted behaviours before and during the lockdown. Important fluctuations were found in all dimensions during the lockdown, and children and adolescents with previous low severity levels of these behaviours significantly worsened during the lockdown, but those with moderate and severe levels showed important improvement during the lockdown [32].

It is important to appreciate the likely consequences of repeated lockdowns or periods of self-isolation and uncertainty around future restrictions on the mental health of young people with ADHD alongside the impact of a less agile economy on the family unit [27]. A recent narrative review found that during confinement, children and adolescents with ADHD reported increased behavioural and ADHD-related symptoms and overall decreased psychological well-being. Less physical activity, adverse parental behaviour, difficulties in coping with preventive COVID-19 guidelines, and school closure and remote learning consequences negatively impacted ADHD-related symptoms and decreased psychological well-being [17].

Reduced cognitive and social demands, and more readily available resources in the home environment during the COVID-19 period resulted in increased home participation in ADHD children compared to the pre-COVID-19 period [28], and parents reported positive family time with their children with ADHD [53]. However, youth with ADHD were less responsive to protective environmental variables (e.g., parental monitoring, school engagement) during the pandemic and may need more specialized support with a return to in-person schooling and daily activities [47].

In terms of impact on parents, psychological distress and burden have been reported to have increased in caregivers of children with and without special educational needs and/or neurodevelopmental differences (SEN/ND) six months into the COVID-19 pandemic, with greater challenges for caregivers of children suffering from ADHD or autism spectrum disorders (ASD), although they seem to be somewhat more resilient compared to caregivers of children without developmental disabilities (DD) [27]. ADHD is associated with poorer outcomes in COVID-19 infection, and untreated ADHD seems to constitute a risk factor for COVID-19 infection while drug treatment reduces this effect [33]. Emerging data have also suggested that mental health complications can occur following COVID-19 infection [62, 67]. In an analysis of 40,469 adult COVID-19 patients between January and June 2020, about 9,000 patients presented with additional psychiatric symptoms, with the most frequent psychiatric manifestations being anxiety and other related disorders (4.6%), followed by mood disorders (3.8%) and suicidal ideation in about 0.2% of subjects [36], indicating that these symptoms are not specific to only individuals with ADHD. However, for some, long-COVID symptoms include cognitive impairment and memory loss, as well as disturbed sleep and increased anxiety, which can be mistaken for ADHD symptoms and will in the future require accurate diagnosis and treatment.

## Core components of ADHD assessment

The aim of this section is to remind the reader of the core elements of an ADHD assessment as described in clinical guidelines such as those from the European ADHD Guidelines Group (EAGG) [63] and the UK National Institute of Healthcare and Clinical Excellence (NICE) guidelines (NICE 2018). A full clinical and psychosocial assessment should include a discussion about behaviour and symptoms of concern in the different domains and settings of the person’s everyday life; a full developmental and psychiatric history and screen for symptoms; observer reports and assessment of the person’s mental state and global functioning (NICE 2008, 2018). The purpose of the assessment is not only to determine whether the patient has ADHD but also to consider differential and co-occurring disorders as well as formulating the case focusing on medical, cognitive, psychosocial, and wider environmental predisposing and maintaining factors and protective influences. It should also evaluate the risk of harm and distress to and from the child or adolescent. To achieve this, in all cases, diagnostic assessment must include: (i) a detailed history-taking from parents/caregivers of development and current state as well as medical, psychiatric, and family history; (ii) obtaining collateral information about symptoms in other contexts such as school; and (iii) direct interview, including observation, with the child or adolescent. Assessment should consider the developmental level of the patient, (which may differ from chronological age) for example, early childhood, middle childhood, and adolescence, and ensure that the reported symptoms are inappropriate for their developmental level. However, the pandemic may have wider impacts on the attainment of age-expected trajectories and milestones due to challenges arising from online schooling, adjusting to going back to school, impact of missed curricula, and for those who had their whole school history during the in-pandemic period. Typically, with ADHD, impairment will exist independently of the pandemic. Effects of lockdown can worsen pre-existing symptoms and impairment. Patients who develop ADHD symptoms solely during lockdown should be re-assessed when lockdown restrictions have been lifted, with the standard criteria for the duration (persistence) and pervasiveness still being applied. Cognitive and/or language assessments may be necessary for some individuals with suspected specific learning or language disability, or intellectual disability. History taking about medical conditions is mandatory and physical/neurological examination is usually necessary before initiating treatment, to identify neuro-genetic disorders, and (rare) physical causes as well as identifying any possible contraindications to medication such as cardiac problems.

## Remote ADHD Diagnostic Assessment in the COVID-19 era

### Published guidance

During the pandemic, remote diagnostic assessment and management of ADHD during the pandemic have been facilitated by rapid publication guidance, such as those from the European ADHD Guidelines Group (EAGG) [15, 16] and the Canadian ADHD Resource Alliance [10]. These sources provide useful information on approaches that could be considered when making a remote assessment. It is recommended that clinicians conducting telehealth work follow the digital guidance from official bodies such as the Royal College of Psychiatrists (https://www.rcpsych.ac.uk/about-us/responding-to-covid-19/responding-to-covid-19-guidance-for-clinicians/digital-covid-19-guidance-for-clinicians) (see Table 1). Remote ADHD assessments should be adapted in line with national and international evidence-based guidelines and practice recommendations as described above. Even though one is doing an ADHD assessment remotely, we recommend that all required components of assessment as summarized above should be completed although may be adapted.

**Table 1:** The digital health guidance from the Royal College of Psychiatrists, UK

Operational issues for those undertaking remote consultations			
Access to technology	Problems with accessibility due to disability	Availability of a carer to assist	Avoid generalization and personalise at a case by case level
Ensure the patient has access to the technology they require, including internet access, as well as the skills to use it—check this and provide a quick training session of how to use the video chat if necessary	Consider any problems with accessibility, in terms of hearing loss or difficulties with dexterity due to coordination problems or arthritis	Plan the video consultation when there may be a carer who can help the patient if they have difficulties in doing this	Avoid generalisation about any specific group in terms of whether video consultation may or may not be suitable. Consider on a case by case, using an understanding of the patient’s needs and circumstances

The 6 “C’s” useful in professionals’ own approach and development when considering video-consultations		
Competence	Communication	Contingencies
Take time to develop enough competence in the platform you intend to use	Consider how to adapt your communication skills to improve the experience. Where the patient is new to you—take even more time over the introduction and signpost what is going to happen next	Have a clear understanding of what to do when the consultation is not going well for technical or clinical reasons
Familiarise yourself with the video consultation platform available to you, and ensure you understand what all the "buttons" or options do	Try to allow for as much non-verbal communication to be captured as possible. Including your head, neck, upper body and arms may be better than just your head. Encourage patient do the same	Have a backup plan for managing any technical difficulties (e.g., loss of connection) and provide this via email to the patient ahead of the session for example or in the first few minutes of the call. Check you have the right mobile telephone number to call them as a backup. Agree who will contact whom in the event of a lost connection
Test the use of the platform and its features with a colleague who may be working from home	Lighting and background are important—plain, darker static/uncluttered background with light directly on your face may help, particularly where the connection is of lower quality	Brief the patient that if you do not feel able to complete an adequate assessment you will discuss what steps to take next. This will include reviewing the risks of a face-to-face contact in the context of the local recommended social distancing policy and delay in care that might result
Make a note of the features you might want to use and have a crib sheet available to you in case you need to refer to it quickly	Slow the rate of speech to allow for problems with slow connections and pause between sentences longer than you might do face-to-face	Ideally have this process mapped out in front of you until you are familiar with it
	Use clear language—again this may be shorter than in face-to-face to ensure clarity of expression across the video call	Practise the “script” that you might want to use for managing contingencies and ensure that the description of how to manage the “what ifs” are clear
	To establish eye contact, you need to look at the camera, not at the eyes of the person you are interviewing	Make sure the technology (laptop, phone) is charged or plugged in and advise, where possible, the patient does the same. If possible, have a backup device available
	Use any features, such as a shared “white board” function, you are familiar with to help with sharing of information	

Confidentiality	Consent	Confidence
If the patient is new to you, verify they are the right person where possible, and check they are expecting the appointment for their mental health and not another condition or problem	As with all appointments, it is important to consider the relevant aspects for meaningful consent	In addressing the other “C’s” you can build the critical sense of confidence for you and the patient in using video consultations
Check who is in the room with the patient, ask for them to be introduced to you, and if possible/practical that they remain in view	Be clear with the patient on the limitation of the assessment or review, and whether they have any concerns	Being confident about using the technology, having a clear sense of what to do if something goes wrong and what the limitations of the technology are will help you develop a confident approach to using video consultations
If the patient is in a public place (e.g., on the bus), consider with them whether it is appropriate to continue at that time. If the need to be in a public place to make use of a public connectivity, explore whether they can move to a private room or consider alternative arrangements	Ensure that you are clear about the security of the platform you are using and that it is fit for purpose to be used for such an encounter and be able to discuss this with the patient if they require. Your local IT training/support team will help you with this	You need to be confident that you have been able to a do a good enough review or assessment. If you have not been able to, do not be afraid to say this, and develop a clear plan of what you need to do next with patient—just like you would in a face-to-face consultation. Whilst under pressure to continue to deliver services against significantly unusual circumstances, this remains the most important factor in safe care and discussing this with the patient is key to them having confidence too
Manage your own environment. avoid sensitive, personal details in the background. Lock the door to the room you are in, if possible, to avoid disruption	Ensure that you discuss with the patient about recording the session—ask about the use of this recording and ideally agree what might be useful for them to be able to take away and that it will only be for private use	
Some platforms have a function that will blur the background behind you—be familiar with how to enable this		
Have a dedicated clinical account, if you use the video platform socially as well as professionally		
Does the patient have anyone else present in the room (such as a relative/carer/advocate)? If so, allow them to introduce themselves and clarify the purpose of the interview with them		

### Recommendations for remote assessment

Remote assessment should be multidisciplinary, with input from parents/caregivers, children and young people, education and, where appropriate, social care. To assist in remote assessment, multiple platforms could be leveraged to obtain information previously gathered by paper and pencil measures. Systems such as the web-based survey tools Qualtrics (https://www.qualtrics.com) and the Research Electronic Data Capture (REDCap) [23] and other web-based platforms such as the HealthTracker™ [50, 51] or the Hogrefe Testsystem (https://www.hogrefe.com/us/testsystem) can be used in several healthcare settings in different countries. It is, however, important to ascertain whether the system selected has the ease with which routine care providers can obtain and engage with it and meets the appropriate privacy-compliance and level of confidentiality requirements necessary in the country of use. Providers should be aware of the impact that different technology platforms can have on patient-clinician rapport and communication. Remote assessments are typically supported by additional communication technologies such as e-mailed consultation information, patient portals, telephone, mobile devices, and electronic health records. Providers should have clear policies pertaining to communications with patients, which describe the boundaries around ways in which patients can communicate with a provider, using appropriate content that can be shared over different technology platforms, anticipated response times, and how and when to contact a provider [55].

Wherever possible, it is important to offer choice in terms of how an assessment is conducted. It is possible to mix and match components, with some aspects (for example, parental history-taking) being done remotely using videoconferencing (as opposed to telephone calls) and others (for example, meeting with the child or the young person) done in person, allowing direct observation. While online communication is increasingly familiar to the public, some families will continue to need support in using videoconferencing tools. We recommend enquiring about this in advance of the assessment. Disruptions in video conferencing technology would need to be considered when carrying out remote assessments as not all young people, their families or carers will have access to high-speed internet and cameras or the privacy needed for assessment. Both parents/caregivers and children require private space to be able to discuss things openly. Access to care and privacy and confidentiality may be more difficult in the overcrowded homes of disadvantaged families [13]. Moreover, such a virtual environment may not be as rewarding as the one that families and carers would otherwise get during face-to-face clinical visits [2], although the experience of assessment done face-to-face because masks may not be well accepted by children, young people and their families. Some individuals, especially those with communication problems may find it overwhelming to video-call, but these can often be identified with the pre-assessment courtesy call and modifications put in place. Some individuals may prefer remote assessments, while others may find it more distracting than face-to-face meetings because of other factors (phones, things in their room) and may find it uncomfortable to share emotional content (e.g., depressive, suicidal thoughts) remotely, and importantly, providing appropriate and timely support may be difficult remotely if this is happening. For other patients and families, the advantages of telehealth in avoiding travel time, costs for travel or loss of parental earnings due to taking time off for the in-person assessment and challenges around travelling with hyperactive and impulsive children are considerable.

A recent study investigated whether telemedicine/telehealth were preferable to face-to-face consultations for 117 adults referred to an NHS Autism and ADHD Service during the COVID-19 pandemic from April to September 2020 [69]. Patients reported that they found telemedicine/telehealth to be useful, effective, reliable, and satisfactory. There was no difference in the choice of contact between the ADHD and Autism pathways. Despite this, almost half of the service users stated a general preference for face-to-face consultations [69].

### Information gathering from teachers

Information from teachers should include contextual information such as percentage rate of school attendance, periods of remote or virtual teaching, periods of lockdown, and degree of engagement in education, as it may be difficult to judge a pupil’s attention, activity level and impulsivity under these circumstances. School information can be gathered using a telephone interview such as the CHATTI [25]. The validity of information from schools (including teacher-reported ADHD questionnaires) in periods of remote or virtual schooling is uncertain and has not been formally assessed. It might be difficult to differentiate who is presenting with ADHD symptoms because individuals may have “adapted” to a complex situation, from those who have neurodevelopmental symptoms and would have presented with similar difficulties anyway. With home schooling, the duration of teacher observations was reduced [64] and occured in a different context to the classroom. As a result, standard teacher reports may be either partial or missing. When necessary, one can use parental/caregiver reports about the quality of engagement in remote or virtual learning during periods when teaching is totally virtual but getting an additional independent report from teachers as soon as possible would be appropriate. However, there is a need to systematically examine whether remote assessments lead to increased rates of mis-, under- or overdiagnosis.

A pilot project evaluated a school-based ADHD Telemedicine Clinic’s adherence to AAP guidelines using chart data before the COVID-19 pandemic [40]. The study included 22 patients (Mean age = 9.3 years, SD = 2.3 years) participating in 69 telemedicine visits across 13 different school-related sites. The ADHD Telemedicine Clinic achieved extremely high adherence rates across the AAP evaluation guidelines for ADHD, ranging from 95 to 100% across the six guidelines. No factor inherent to the telemedicine service delivery mechanism impeded adherence to national guidelines for ADHD evaluation. The school-based telemedicine clinic allowed increased communication across the school and specialty mental health systems and facilitated greater input across child, parent, school personnel, and mental health professionals [40].

### Conducting complex assessments

Where cognitive or language evaluation is deemed important in the diagnostic process, assessments such as *Q Global*, *Q Interactive* or the *Hogrefe Testsystem* are feasible, but patients may require considerable support to use them. One often requires two monitors and a cooperative participant. In clinic settings, such assessments provide an additional opportunity for direct observation, which is not possible during remote delivery of care. Parent ‘coaching’, often aimed at increased compliance, or environmental distractions can limit confidence in interpretation.

### Conducting physical assessments

Basic physical measurements such as height and weight, pulse and blood pressure measurements are important as part of the assessment to rule out non-psychiatric medical factors, and as a baseline when considering medication. Families are usually able to report on height and weight, but pulse and blood pressure measurements may also need to be obtained by training families in the use of a digital blood pressure monitor. It is especially important to recognize that many families may not have a paediatric blood pressure cuff available at home. The general advice is to use age-adapted cuff size, take the BP at least 2 h after taking a dose, sit down for 10–15 min before taking the reading, taking the reading on the left arm, and take the lowest of three readings, approximately the same time on three separate days and send the readings, alongside the pulse values, to the prescribers [14].

### Impact of telemedicine/telehealth

The psychological aspects of using a virtual environment and the lack of in-person contact in a physical therapeutic setting, alongside the potential loss of engagement, also need consideration. This lack of engagement could also pose problems for the quality of the diagnostic assessment. In addition, remote assessments can make it more difficult to identify child abuse and related ADHD-like symptomatology, self-harm, and tics, and hence one needs to have a low threshold for bringing patients in for a face-to-face assessment if one suspects child abuse or maltreatment. On the other hand, the lower cost (no need for transportation, parking, less time off work and off school) and higher flexibility (allowing adaptation to parents work schedules, ability to access care from far away distance) of Telemedicine/Telehealth may be an advantage, as the alternative may be no care, especially for underserved areas far from large metropolitan areas [35].

## Novel/supplementary digital assessment tools/approaches

The various digital health strategies that have been proposed to help ADHD assessment and management are described below:

- **Remote triaging:** Despite limited validation of digital completions of generic symptom questionnaires (e.g., SDQ, CBCL, PONS) and ADHD-specific measures (e.g., SNAP, SWAN, Vanderbilt, ADHD-RS, Conners’, ADHD ratings from the DISYPS system), cut-off scores may assist in identifying patients that require a detailed ADHD assessment [34].
- **Diagnostic assessment:** Digitally completed referral forms for specialist ADHD assessment can assist uniformity and could help reduce time to specialist assessments. A diagnosis of ADHD should not be made solely based on rating scales or observational data. However, ADHD-specific rating scales such as those listed above can be valuable adjuncts in establishing a baseline in terms of the severity of the symptoms, which can be tracked longitudinally, though one must understand the sensitivity and specificity of the measures. Sections of the Diagnostic Interview for Children and Adolescents–Revised (DICA–R), the Diagnostic Interview Schedule for Children (DISC), the Kiddie Schedule for Affective Disorders and Schizophrenia (K–SADS), and the Child and Adolescent Psychiatric Assessment (CAPA) can assist in getting detailed systematic information about ADHD. Remote observations at home and while accessing remote education may be useful when there is doubt about ADHD symptoms. To supplement clinical diagnosis, different online platforms and ADHD assessment tools are available—these are not necessarily available in all languages but have been provided as examples. Completion of the ADHD and related modules of the Development and Well-Being Assessment (DAWBA) which can be completed by multiple informants (parents, teachers, and young people [if age appropriate]), including free text enquiries, and following clinician review, can often provide an accurate diagnostic assessment of ADHD [21] and co-morbid conditions. However, this is not an interview-based assessment, and does not allow for clarification by the clinician with the patient/parent, that is often needed, particularly where co-occurring conditions and/or alternative neurodevelopmental disorders are being considered. The HealthTracker platform ADHD modules can be completed remotely by multiple raters (patient, parent/carer/partner, teachers, employers etc.). It includes ratings of impairment, automatically scored; its results are intuitively presented (https://www.healthtracker.co.uk) and be used as would have been done previously with paper-based questionnaires. To supplement clinical diagnosis, the utility of the computerised QbTest for attention and activity (which must be done face to face, with clinician observation) has been explored [22, 26]. One trial of QbTest to augment standard clinical assessment, reduced the time taken to diagnostic decision regarding ADHD [24]. However, the sensitivity and specificity have not been established for QbTest and it may be used to augment standard clinical assessment and not as a stand-alone diagnostic tool for ADHD.
- **Nodal ADHD re-assessment:** A nodal ADHD re-assessment is undertaken when a patient is coming up to the age of the cut-off boundary for adult services. The purpose is to establish the need for continuing care and treatment into adulthood. Assessment at this point should consider personal, educational, occupational, and social functioning, and include an assessment for coexisting conditions, especially drug misuse, personality disorders, emotional problems and learning difficulties. The Transition Readiness Assessment Measure (TRAM), a web-based measure for transition readiness in young people in CAMHS, has been designed to guide transition planning and was developed as part of the Managing the Link and Strengthening Transition from Child to Adult Mental Health Care (MILESTONE) Project, which focussed on the transition of patients (including those with ADHD) from CAMHS to Adult Mental Health Services [51]. It may be useful to review the diagnosis again at the first face-to-face meeting when the initial diagnosis was conducted remotely, especially in those patients where the clinicians have had some doubt or need extra cognitive assessments to rule out learning disabilities or intellectual disability.
- **Follow-up symptoms and post-treatment monitoring:** Information can be obtained using standardized questionnaires, through ecological momentary assessments by smartphones and/or digital platforms that can capture symptom change, side effects, QoL, impairment, and participation measures. The Children’s ADHD Telemental Health Treatment Study (CATTS) was a randomized controlled trial done in seven communities in Washington and Oregon, that demonstrated the superiority of a telehealth service delivery model for the treatment of ADHD with combined pharmacotherapy and behaviour training (*n* = 111) in reducing distress in caregivers of children with ADHD, compared with management in primary care augmented with a telepsychiatry consultation (*n* = 112). A diagnosis of ADHD was established with the Computerized Diagnostic Interview Schedule for Children (CDISC), and comorbidity for oppositional defiant disorder (ODD) and anxiety disorders (AD) was established using the CDISC and the Child Behaviour Checklist. Telepsychiatrists used the Texas Children’s Medication Algorithm Project (TCMAP) for ADHD to guide pharmacotherapy. Psychoeducation, and caregiver behavioural training were also provided. The study showed that ADHD could be managed remotely in communities with poor access to specialised mental health services [35] and that effective pharmacotherapy for ADHD can be provided [46], with improved outcomes [66]. Existing literature supports remote behavioural health interventions including behavioural parent training and combination approaches for externalizing disorders as well as cognitive-behavioural based interventions for internalizing disorders [48].

## Current limitations of remote assessment | poor practice

It is important to acknowledge that remote assessments may also have limitations, as it may be more difficult to view the whole family on the screen, observe the parent–child interactions, and to keep the child in view as hyperactivity can lead to the child moving away from the camera, and some individuals may struggle to focus for the whole period. Privacy can be compromised as one cannot be certain about who is in the room, and if children (or one or the other parent) can speak openly and freely [55]. Basavarajappa et al. [3] reported on a survey of 340 psychiatrists regarding the advantages and disadvantages of telemedicine during the pandemic. The responders felt that telepsychiatry would provide easy accessibility to mental health services (83.24%), would lead to less exposure to infections (65.29%) and increased doctor shopping resulting in poor care (52.06%). However, they also pointed out that there would be problems dealing with suicidal or homicidal patients (72.06%), and were worried that the patient may record the consultation, leading to legal issues (36.56%) [3].

Table 1 gives details about guidelines in relation to remote assessments.

Here are a few examples of practice to be avoided

1. Issues such as lack of secure space for confidential remote assessment, bad lighting, bad microphone, bad visualization, and audio, in general, can affect optimal remote interactions.
2. Screen distractions, such as having emails flash or arrive at the same time can compromise confidentiality.
3. Patients and families not being informed about security settings of the remote assessment—it is important to obtain participant/informant agreement/consent to proceed with the remote interview.
4. Recording sessions without the knowledge of the other party (patient or clinician/provider), or not informing patient about the presence of others who are not visible on the screen.
5. Using the same link for different patients can lead to breach of confidentiality and unacceptable practice.
6. Performing telemedicine services while the patient is in a moving vehicle, for safety and liability reasons.
7. In the US and many other countries, clinicians and providers should be aware of geographic limitations of their practice based on local licensure laws if seeing patients via Telemedicine.

## Future directions

ADHD assessments can be implemented remotely, most often maintaining high standards. There is a scarcity of research on assessment via telehealth and establishing evidence-based assessment via telehealth will be of increased importance [48]. Differential impact of remote assessment in children versus adolescents will need to be examined. Rating scales that are available for remote administration using protected Apps/Devices are appropriate for use during telehealth evaluation, but future work is needed examining their psychometric properties when administered remotely.

Moreover, there are challenges to overcome and improvements that need to be made. For example, physical assessment, including cardiovascular assessment remains a challenge when in-person visits are disrupted due to lockdowns. Before initiating pharmacotherapy for ADHD, vital signs such as blood pressure and heart rate, alongside height and weight, are routinely measured. We have provided some recommendations for starting medications during the pandemic [15]. As indicated by others [30], it might be prudent to encourage the use of blood pressure monitors or contact photoplethysmography using mobile devices. These technologies could assist in the clinical decision-making process when a diagnosis has been made and before tele-prescribing has been initiated.

Future studies should collect data on patient/family preferences and feedback regarding remote assessment for ADHD and other disorders. Identifying characteristics of patients, families, and clinicians who prefer remote assessments versus face-to-face assessments could help match the type of assessment to appropriate patient and clinician. Also, there is a need to collect health economic data to identify whether remote assessments lead to more cost-effective and/or faster response to patient need. Enhanced telemedicine and remote assessment training will need to become part of routine workforce training.

Exposure to, and infection with, SARS-CoV-2 and its potential short and long-term impacts on ADHD and other mental health disorders has not been adequately explored and more evidence is needed to understand how viral exposure causes short term changes in mental health and what the longer-term impact of this will be. Moving forward in the post-COVID era, remote diagnostic assessment of ADHD for young people can form a vital component of the ADHD assessment ecosystem. However, as clinicians, we would need to be cognisant of the challenges of working in a virtual world, especially when it comes to clinical decision-making for young people with ADHD. Barriers to the use of telehealth platforms include the impact of the complexity and symptoms of illness, lack of face-to-face training, and clinician preference for training mediums [65]. Despite these concerns, remote diagnostic assessment of ADHD in young people using telehealth has the potential of opening new opportunities for assessing, managing, and treating ADHD. Web-based assessments could facilitate diagnosis and treatment monitoring, and telemedicine could continue to inform ADHD practices and guidelines once the restrictions begin to loosen. A hybrid model that implements web-based and semi-structured approaches in a clinical setting could be the way forward for facilitating ADHD diagnosis and management in young people. Successful implementation of such models in medicine may require ongoing organizational support, in-house clinical supervision, and development of tailored e-health resources, and open training opportunities on how to operate and effectively utilize these resources [65].

## Conclusions

Taking together all the above considerations we may conclude:

- With appropriate adaptations, ADHD assessments can be undertaken either remotely or by using personal protective equipment for in-person work. It is important that we continue to complete standard components of ADHD assessment.
- The preferences of patients, families and clinicians should be considered when deciding which approach to take.
- The clinician will have insight into the need for a face-to-face assessment for a particular individual, knowledge about the current covid-related risks to patients and clinicians, both of which should be considered when deciding on appointment type.
- If a traditional face-to-face assessment cannot be done due to restrictions, ADHD assessment should not be delayed and a remote assessment should be considered.
- Clinicians should be aware that ADHD may be a risk factor for difficulty complying with social and related restrictions, and this should be addressed during the diagnostic assessment. Such restrictions, including changes in education can either mask or exacerbate features associated with ADHD and, therefore, assessment needs to carefully chart symptomatic manifestations prior to as well as during the pandemic.
- Experience with remote ADHD assessment may also provide a useful template/paradigm for the assessment of other childhood psychopathology.
- While remote assessments are valuable in allowing work to continue despite restrictions and may have benefits for routine care in the post-pandemic world, it is important to ensure access for groups who are digitally disadvantaged. Those who may not be able to use/access remote technologies should be prioritized for conventional or other alternative assessments.
- It is likely that the delivery of medical care will evolve and involve blended models of needs-based face-to-face and remote contacts.

## Abbreviations

ADHD: Attention-deficit/hyperactivity disorder
ADHD-RS: ADHD rating scale
ASD: Autism Spectrum Disorders
CAMHS: Child and Adolescent Mental Health Services
CBCL: Child Behavior Checklist
Conners: Conners rating scale for ADHD
DAWBA: Development and Well-Being Assessment
DD: Developmental disabilities
DISYPS: Diagnostik-System für psychische Störungen nach ICD-10 und DSM-5 für Kinder- und Jugendliche (DISYPS-III)
EAGG: European ADHD Guidelines Group
NICE: UK National Institute of Healthcare and Clinical Excellence
PCPs: Primary care providers
PONS: Profile of Neuropsychiatric Symptoms
QbTest: Quantified Behavioral Test
QoL: Quality of Life
SDQ: Strengths and Difficulties Questionnaire
SEN/ND: Special educational needs and/or neurodevelopmental differences
SNAP: SNAP-IV rating scale
SWAN: SWAN rating scale for ADHD
TRAM: Transition Readiness and Appropriateness Measure
Vanderbilt: Vanderbilt ADHD Diagnostic Rating Scale

## References

[1] Agnew-Blais JC, Polanczyk GV, Danese A, Wertz J, Moffitt TE, Arseneault L (2018). Young adult mental health and functional outcomes among individuals with remitted, persistent and late-onset ADHD. Br J Psychiatry.

[2] Aman GM, Pearson AD (2020). Challenges for child and adolescent psychiatric research in the era of COVID-19. J Child Adolesc Psychopharmacol.

[3] Basavarajappa C, Grover S, Dalal PK, Avasthi A, Kumar CN, Manjunatha N, Sahoo S, Saha G, Mehra A, Singh OP, Tripathi A, Gangadhar BN, Math SB (2022). Perceived advantages and disadvantages of telepsychiatry—an online survey of psychiatrists in India. Indian J Psychiatry.

[4] Becker SP, Breaux R, Cusick CN, Dvorsky MR, Marsh NP, Sciberras E (2020). Remote learning during COVID-19: examining school practices, service continuation, and difficulties for adolescents with and without attention-deficit/hyperactivity disorder. J Adolesc Health.

[5] Bemanalizadeh M, Yazdi M, Yaghini O, Kelishadi R (2021). A meta-analysis on the effect of telemedicine on the management of attention deficit and hyperactivity disorder in children and adolescents. J Telemed Telecare.

[6] Bobo E, Lin L, Acquaviva E, Caci H, Franc N, Gamon L (2020). Comment les enfants et adolescents avec le trouble déficit d’attention/hyperactivité (TDAH) vivent-ils le confinement durant la pandémie COVID-19? [How do children and adolescents with Attention Deficit Hyperactivity Disorder (ADHD) experience lockdown during the COVID-19 outbreak?]. Encephale.

[7] Breaux R, Dvorsky MR, Marsh NP, Green CD, Cash AR, Shroff DM, Buchen N, Langberg JM, Becker SP (2021). Prospective impact of COVID-19 on mental health functioning in adolescents with and without ADHD: protective role of emotion regulation abilities. J Child Psychol Psychiatry.

[8] Bruni O, Giallonardo M, Sacco R, Ferri R, Melegari MG (2021). The impact of lockdown on sleep patterns of children and adolescents with ADHD. J Clin Sleep Med.

[9] Buchen N, Langberg JM, Becker SP (2021). Prospective impact of COVID-19 on mental health functioning in adolescents with and without ADHD: protective role of emotion regulation abilities. J Child Psychol Psychiatry.

[10] CADDRA (2020) ADHD and COVID-19 frequently asked questions. https://www.caddra.ca/wp-content/uploads/CADDRA-ADHD-and-Virtual-Care-FAQ.pdf. Accessed 30th Apr 2022

[11] Chandra P, Kar N, Yahia A (2021). Experience of tele-psychiatry during COVID-19 amongst doctors working in a mental health trust: a survey. BJPsych Open.

[12] Coghill D, Banaschewski T, Cortese S, Asherson P, Brandeis D, Buitelaar J, Daley D, Danckaerts M, Dittmann RW, Doepfner M, Ferrin M, Hollis C, Holtmann M, Santosh P, Sonuga-Barke E, Soutullo C, Steinhausen HC, Van der Oord S, Wong ICK, Zuddas A, Simonoff E (2021). The management of ADHD in children and adolescents: bringing evidence to the clinic: perspective from the European ADHD Guidelines Group (EAGG). Child Adolesc Psychiatry.

[13] Corso PS, Visser SN, Ingels JB, Perou R (2015). Cost-effectiveness of legacy for children™ for reducing behavioral problems and risk for ADHD among children living in poverty. J Child Adolesc Behav.

[14] Cortese S, Asherson P, Sonuga-Barke E, Banaschewski T, Brandeis D, Buitelaar J (2020). European ADHD Guidelines Group. ADHD management during the COVID-19 pandemic: guidance from the European ADHD Guidelines Group. Lancet Child Adolesc Health.

[15] Cortese S, Coghill D, Santosh P, Hollis C, Simonoff E (2020). Starting ADHD medications during the Covid-19 pandemic. Recommendations from the European ADHD Guidelines Group (EAGG). Lancet Child Adolescent Health.

[16] Cortese S (2020). Pharmacologic treatment of attention deficit-hyperactivity disorder. N Engl J Med.

[17] Davoody S, Goeschl S, Dolatshahi M, Davari-Ashtiani R, Saffarpour R, Sodeifian F, Brand S (2022). Relation between ADHD and COVID-19: a narrative review to guide advancing clinical research and therapy. Iran J Psychiatry.

[18] Döpfner M, Görtz-Dorten A (2017). Diagnostik-System für psychische Störungen nach ICD-10 und DSM-5 für Kinder- und Jugendliche (DISYPS-III).

[19] Du Rietz E, Brikell I, Butwicka A, Leone M, Chang Z, Cortese S, D'Onofrio BM, Hartman CA, Lichtenstein P, Faraone SV, Kuja-Halkola R, Larsson H (2021). Mapping phenotypic and aetiological associations between ADHD and physical conditions in adulthood in Sweden: a genetically informed register study. Lancet Psychiatry.

[20] Faraone SV, Banaschewski T, Coghill D, Zheng Y, Biederman J, Bellgrove MA, Newcorn JH, Gignac M, Al Saud NM, Manor I, Rohde LA, Yang L, Cortese S, Almagor D, Stein MA, Albatti TH, Aljoudi HF, Alqahtani MMJ, Asherson P, Atwoli L, Bölte S, Buitelaar JK, Crunelle CL, Daley D, Dalsgaard S, Döpfner M, Espinet S, Fitzgerald M, Franke B, Gerlach M, Haavik J, Hartman CA, Hartung CM, Hinshaw SP, Hoekstra PJ, Hollis C, Kollins SH, Sandra Kooij JJ, Kuntsi J, Larsson H, Li T, Liu J, Merzon E, Mattingly G, Mattos P, McCarthy S, Mikami AY, Molina BSG, Nigg JT, Purper-Ouakil D, Omigbodun OO, Polanczyk GV, Pollak Y, Poulton AS, Rajkumar RP, Reding A, Reif A, Rubia K, Rucklidge J, Romanos M, Ramos-Quiroga JA, Schellekens A, Scheres A, Schoeman R, Schweitzer JB, Shah H, Solanto MV, Sonuga-Barke E, Soutullo C, Steinhausen HC, Swanson JM, Thapar A, Tripp G, van de Glind G, Brink WVD, Van der Oord S, Venter A, Vitiello B, Walitza S, Wang Y (2021). The World Federation of ADHD international consensus statement: 208 evidence-based conclusions about the disorder. Neurosci Biobehav Rev.

[21] Foreman D, Morton S, Ford T (2009). Exploring the clinical utility of the Development And Well-Being Assessment (DAWBA) in the detection of hyperkinetic disorders and associated diagnoses in clinical practice. J Child Psychol Psychiatry.

[22] Hall CL, Valentine AZ, Walker GM, Ball HM, Cogger H, Daley D (2017). Study of user experience of an objective test (QbTest) to aid ADHD assessment and medication management: a multi-methods approach. BMC Psychiatry.

[23] Harris PA, Taylor R, Thielke R, Payne J, Gonzalez N, Conde JG (2009). Research electronic data capture (REDCap)—A metadata-driven methodology and workflow process for providing translational research informatics support. J Biomed Inform.

[24] Hollis C, Hall CL, Guo B, James M, Boadu J, Groom MJ (2018). the AQUA Trial Group. The impact of a computerised test of attention and activity (QbTest) on diagnostic decision-making in children and young people with suspected attention deficit hyperactivity disorder: single-blind randomised controlled trial. J Child Psychol Psychiatry.

[25] Holmes J, Lawson D, Langley K, Fitzpatrick H, Trumper A, Pay H, Harrington R, Thapar A (2004). The child attention-deficit hyperactivity disorder teacher telephone interview (CHATTI): reliability and validity. Br J Psychiatry.

[26] Hult N, Kadesjö J, Kadesjö B, Gillberg C, Billstedt E (2015). ADHD and the QbTest: diagnostic validity of QbTest. J Attention Disord.

[27] Iovino EA, Caemmerer J, Chafouleas SM (2021). Psychological distress and burden among family caregivers of children with and without developmental disabilities six months into the COVID-19 pandemic. Res Dev Disabil.

[28] Kaya Kara O, Tonak HA, Kara K, Sonbahar Ulu H, Kose B, Sahin S, Kara MZ (2021). Home participation, support and barriers among children with attention-deficit/hyperactivity disorder before and during the COVID-19 pandemic. Public Health.

[29] Magson NR, Freeman JYA, Rapee RM, Richardson CE, Oar EL, Fardouly J (2021). Risk and protective factors for prospective changes in adolescent mental health during the COVID-19 pandemic. J Youth Adolesc.

[30] McGrath J (2020). ADHD and Covid-19: current roadblocks and future opportunities. Ir J Psychol Med.

[31] Meherali S, Punjani N, Louie-Poon S, Rahim KA, Das JK, Salam RA, Lassi ZS (2021). Mental health of children and adolescents amidst COVID-19 and past pandemics: a rapid systematic review. Int J Environ Res Public Health.

[32] Melegari MG, Giallonardo M, Sacco R, Marcucci L, Orecchio S, Bruni O (2020). Identifying the impact of the confinement of Covid-19 on emotional-mood and behavioural dimensions in children and adolescents with attention deficit hyperactivity disorder (ADHD). Psychiatry Res.

[33] Merzon E, Weiss MD, Cortese S, Rotem A, Schneider T, Craig SG, Vinker S, Golan Cohen A, Green I, Ashkenazi S, Weizman A, Manor I (2022). The association between ADHD and the severity of COVID-19 infection. J Atten Disord.

[34] Mulraney M, Arrondo G, Musullulu H, Iturmendi-Sabater I, Cortese S, Westwood SJ (2021). Systematic review and meta-analysis: screening tools for attention-deficit/hyperactivity disorder in children and adolescents. J Am Acad Child Adolesc Psychiatry.

[35] Myers K, Vander Stoep A, Zhou C, McCarty CA, Katon W (2015). Effectiveness of a telehealth service delivery model for treating attention-deficit/hyperactivity disorder: a community-based randomized controlled trial. J Am Acad Child Adolesc Psychiatry.

[36] Nalleballe K, Reddy Onteddu S, Sharma R (2020). Spectrum of neuropsychiatric manifestations in COVID-19. Brain Behav Immun.

[37] National Institute for Health and Care Excellence (2008) Attention deficit hyperactivity disorder: diagnosis and management. NICE Guideline CG72. https://www.nice.org.uk/guidance/cg72 29634174

[38] National Institute for Health and Care Excellence (2018) Attention deficit hyperactivity disorder: diagnosis and management. NICE Guideline NG87. https://www.nice.org.uk/guidance/ng87/chapter/recommendations. (Accessed 12 Jan 2021) 29634174

[39] Nearchou F, Flinn C, Niland R, Subramaniam SS, Hennessy E (2020). Exploring the impact of COVID-19 on mental health outcomes in children and adolescents: a systematic review. Int J Environ Res Public Health.

[40] Nelson EL, Duncan AB, Peacock G, Bui T (2012). Telemedicine and adherence to national guidelines for ADHD evaluation: a case study. Psychol Serv.

[41] Panda PK, Gupta J, Chowdhury SR, Kumar R, Meena AK, Madaan P (2020). Psychological and behavioral impact of lockdown and quarantine measures for COVID-19 pandemic on children, adolescents and caregivers: a systematic review and meta-analysis. J Trop Pediatr.

[42] Penner F, Ortiz JH, Sharp C (2021). Change in youth mental health during the COVID-19 pandemic in a majority Hispanic/Latinx US sample. J Am Acad Child Adolesc Psychiatry.

[43] Polanczyk G, De Lima MS, Horta BL, Biederman J, Rohde LA (2007). The worldwide prevalence of ADHD: a systematic review and meta-regression analysis. Am J Psychiatry.

[44] Racine N, McArthur BA, Cooke J, Eirich R, Zhu J, Madigan S (2021). Global prevalence of depressive and anxiety symptoms in children and adolescents during COVID-19. A meta-analysis. JAMA Pediatr.

[45] Raw JAL, Waite P, Pearcey S, Shum A, Patalay P, Creswell (2021). Examining changes in parent-reported child and adolescent mental health throughout the UK's first COVID-19 national lockdown. J Child Psychol Psychiatry.

[46] Rockhill CM, Tse YJ, Fesinmeyer MD, Garcia J, Myers K (2016). Telepsychiatrists' medication treatment strategies in the children's attention-deficit/hyperactivity disorder telemental health treatment study. J Child Adolesc Psychopharmacol.

[47] Rosenthal E, Franklin-Gillette S, Jung HJ, Nelson A, Evans SW, Power TJ, Yerys BE, Dever BV, Reckner E, DuPaul GJ (2021). Impact of COVID-19 on youth with ADHD: predictors and moderators of response to pandemic restrictions on daily life. J Atten Disord.

[48] Ros-DeMarize R, Chung P, Stewart R (2021). Pediatric behavioral telehealth in the age of COVID-19: brief evidence review and practice considerations. Curr Probl Pediatr Adolesc Health Care.

[49] Royal College of Psychiatrists. https://www.rcpsych.ac.uk/about-us/responding-to-covid-19/responding-to-covid-19-guidance-for-clinicians/digital-covid-19-guidance-for-clinicians. Accessed 12 January 2021

[50] Santosh P, Bell L, Fiori F, Singh J (2016). Pediatric antipsychotic use and outcomes monitoring. J Child Adolesc Psychopharmacol.

[51] Santosh P, Singh J, Adams L, Mastroianni M, Heaney N, Lievesley K (2020). Validation of the transition readiness and appropriateness measure (TRAM) for the managing the link and strengthening transition from child to adult mental healthcare in Europe (MILESTONE) study. BMJ Open.

[52] Sayal K, Prasad V, Daley D, Ford T, Coghill D (2018). ADHD in children and young people: prevalence, care pathways, and service provision. Lancet Psychiatry.

[53] Sciberras E, Patel P, Stokes MA, Coghill D, Middeldorp CM, Bellgrove MA (2020). Physical health, media use, and mental health in children and adolescents with ADHD during the COVID-19 pandemic in Australia. J Atten Disord.

[54] Shah R, Raju VV, Sharma A, Grover S (2021). Impact of COVID-19 and lockdown on children with ADHD and their families—an online survey and a continuity care model. J Neurosci Rural Pract.

[55] Shore JH, Yellowlees P, Caudill R, Johnston B, Turvey C, Mishkind M, Krupinski E, Myers K, Shore P, Kaftarian E, Hilty D (2018). Best practices in videoconferencing-based telemental health april 2018. Telemed J E Health.

[56] Shum A, Skripkauskaite S, Pearcey S, Waite P, Creswell C (2021) Report 10: Children and adolescents’ mental health: One year in the pandemic

[57] Sibley MH, Ortiz M, Gaias LM, Reyes R, Joshi M, Alexander D, Graziano P (2021). Top problems of adolescents and young adults with ADHD during the COVID-19 pandemic. J Psychiatr Res.

[58] Song P, Zha M, Yang Q, Zhang Y, Li X, Rudan I, the Global Health Epidemiology Reference Group (GHERG) (2021). The prevalence of adult attention-deficit hyperactivity disorder: a global systematic review and meta-analysis. J Glob Health.

[59] Sonuga-Barke E, Fearon P (2021). Editorial: Do lockdowns scar? Three putative mechanisms through which COVID-19 mitigation policies could cause *long-term* harm to young people's mental health. J Child Psychol Psychiatry.

[60] Sonuga-Barke EJ, Halperin JM (2010). Developmental phenotypes and causal pathways in attention deficit/hyperactivity disorder: potential targets for early intervention?. J Child Psychol Psychiatry.

[61] Swansburg R, Hai T, MacMaster FP, Lemay JF (2021). Impact of COVID-19 on lifestyle habits and mental health symptoms in children with attention-deficit/hyperactivity disorder in Canada. Paediatr Child Health.

[62] Swanson JM, Volkow ND (2021). Lessons from the 1918 flu pandemic: a novel etiologic subtype of ADHD?. J Am Acad Child Adolesc Psychiatry.

[63] Taylor E, Dopfner M, Sergeant J, Asherson P, Banaschewski T, Buitelaar J, Coghill D, Danckaerts M, Rothenberger A, Sonuga-Barke E, Steinhausen H-C, Zuddas A (2004). European clinical guidelines for hyperkinetic disorder—first upgrade. Eur Child Adolesc Psychiatry.

[64] Thorell L, Borg Skoglund C, Gimenez de la Pena A, Baeyens D, Fuermaier A, Groom M, Mammarella I, Van der Oord S, van den Hoofdakker B, Luman M, Marques de Miranda D, Siu A, Steinmayr R, Idrees I, Soares LS, Sorlin M, Luque JL, Moscardino U, Roch M, Crisci G, Christiansen H (2021). Parental experiences of homeschooling during the COVID-19 pandemic: differences between seven European countries and between children with and without mental health conditions. Eur Child Adolesc Psychiatry.

[65] Uribe Guajardo MG, Baillie A, Louie E (2022). The evaluation of the role of technology in the pathways to comorbidity care implementation project to improve management of comorbid substance use and mental disorders. J Multimorb Comorb.

[66] Vander Stoep A, McCarty CA, Zhou C, Rockhill CM, Schoenfelder EN, Myers K (2017). The children's attention-deficit hyperactivity disorder telemental health treatment study: caregiver outcomes. J Abnorm Child Psychol.

[67] Varatharaj A, Thomas N, Ellul MA, Davies NWS, Pollak TA, Tenorio EL (2020). CoroNerve Study Group. Neurological and neuropsychiatric complications of COVID-19 in 153 patients: a UK-wide surveillance study. Lancet Psychiatry.

[68] Waite P, Pearcey S, Shum A, Raw J, Patalay P, Creswell C (2020) How did the mental health of children and adolescents change during early lockdown during the COVID-19 pandemic in the UK? 10.31234/osf.io/t8rfx 10.1111/jcv2.12009 PMC8206715 34485988

[69] Adamou M, Jones SL, Fullen T, Galab N, Abbott K, Yasmeen S (2021). Remote assessment in adults with Autism or ADHD: A service user satisfaction survey. PLOS ONE.

